# Association between monoamine oxidase A promoter polymorphism and the risk of sudden infant death syndrome: a meta-analysis

**DOI:** 10.1007/s00414-020-02496-6

**Published:** 2021-02-01

**Authors:** Qiaoxia Zhou, Daoyin Gong, Yu Zhang, Feijun Huang

**Affiliations:** 1 Department of Forensic Pathology, West China School of Preclinical and Forensic Medicine, Sichuan University, No. 17 Third Renmin Road North, Chengdu, 610041 China; 2 Hospital of Chengdu University of Traditional Chinese Medicine, Chengdu, China; 3 Department of Mechanical Engineering, Technical University of Munich, Munich, Germany

**Keywords:** Sudden infant death syndrome, MAOA, Serotonin, Gene polymorphism, Meta-analysis

## Abstract

**Introduction:**

The etiology of sudden infant death syndrome (SIDS) remains an unsolved problem. The aim of this meta-analysis is to investigate the potential association between monoamine oxidase A (MAOA) promoter variable number tandem repeat (VNTR) polymorphism and SIDS risk.

**Methods:**

A systematic review and meta-analysis were conducted on studies from accessible electronic databases. Each VNTR variant was examined in each gender independently by comparing with the pooled results of other alleles.

**Results:**

A total of six independent case–control studies including 1022 SIDS cases and 1839 controls were enrolled in this meta-analysis. In both of the whole populations and Caucasian populations, male infants with the low-MAOA-expression alleles (2R+3R) were found to exhibit a statistically significant increased risk of SIDS, whereas those with a 4R allele exhibited a reduced risk of SIDS. Besides, an increased risk of SIDS was detected in male Caucasian infants with 2R or 3R alleles. However, none of the allele or genotype variants was associated with SIDS in female victims.

**Conclusion:**

In male Caucasian infants, the low expression of MAOA promoter VNTR alleles (2R and 3R) is associated with an increased risk of SIDS, and the existence of the 4R allele could be regarded as a protective factor.

## Introduction

Sudden infant death syndrome (SIDS) is defined as the sudden unexpected death that occurs during sleep in infants under one year of age, which cannot be explained by integrated forensic investigations, including systematic autopsy, examination of the circumstances, and review of the clinical history [1]. Regarded as one of the leading causes of infant death in developed countries, SIDS is suggested to be a multifactorial disease as its occurrence is probably dependent on the coalescence of three classes of risk factors including predisposing, developmental and environmental [2–4], which was so-called “triple risk hypothesis” and makes the etiology more complex [5, 6]. However, the etiology of SIDS is still unknown, attracting the attention of researchers worldwide [7, 8].

Some progress has been made in molecular genetics in research investigating the pathogenic mechanism of SIDS, breaking the impasse and opening new perspectives in this field. Increasing evidence has shown that autonomic nervous system disturbances may play an important role in the occurrence of SIDS, especially those involved in respiratory regulation and arousal reaction. These impaired functions are associated with an abnormal serotonin level in the brainstem, 5-HT1A receptors, and multiple functional gene polymorphisms of the serotonin transporter, leading to a failure in homeostatic defensive responses to life-threatening problems during sleep [9–11]. In addition to serotonergic neurons, noradrenaline is also one of the neurotransmitters involved in the modulation of respiratory activity and occurrence of SIDS [12] with evidence such as differences in expression of the tyrosine hydroxylase [13] and the identification of a functional polymorphism in the tyrosine hydroxylase gene as a risk factor of SIDS [14].

To further investigate the correlation between neurotransmitter systems and SIDS, a variety of gene polymorphisms have been widely studied [15, 16], including polymorphisms of TH01 in the tyrosine hydroxylase gene, serotonin transporter promoter, and monoamine oxidase A (MAOA) promoter. MAOA, a flavin-adenine-dinucleotide-dependent enzyme, regulates the presynaptic levels of noradrenalin and 5-HT via the deamination and inactivation of monoaminergic neurotransmitters [17]. Polymorphism of the MAOA promoter, located on the X-chromosome 1.2 kb upstream of the MAOA gene, comprising five alleles, that is, 2, 3, 3.5, 4, and 5 copies (2R, 3R, 3.5R, 4R, and 5R) of a 30 bp variable number tandem repeat (VNTR) sequence, was reported to affect the gene transcriptional potential of MAOA [16–18]. Compared with the longer 3.5- and 4-copy alleles (high-expression alleles 3.5R and 4R), the shorter 2- and 3-copy alleles (low-expression alleles 2R and 3R) have been reported to reduce the promoter activity 2–10 times, leading to a higher 5-HT level in the synapses [14]. However, it has been reported in several studies that the transcriptional activity of the rarely presenting allele 5R is different; Deckert et al. [19] claimed that the expression activity is high, whereas Sabol et al. [18] suggested lower expression activity.

Considering that SIDS has been reported to be associated with abnormalities of neurotransmitter systems, MAOA promoter polymorphism, which could regulate the levels of serotonin and noradrenalin, is considered to be of great potential to be correlated with the risk of SIDS. However, no consensus regarding the VNTR polymorphism of the MAOA promoter and the incidence of SIDS has yet been reached in various studies. Filonzi et al. [20] demonstrated that the 3R allele with low transcriptional activity is associated with a higher risk of SIDS compared to alleles with high transcriptional activity, whereas Opdal et al. [21] indicated that there is no correlation between the VNTR polymorphism of MAOA and SIDS. Regarding differences between genders, the findings of previously published studies have also been inconclusive. Courts et al. [22] detected a significant difference among allelic and genotypic variants between female SIDS victims and controls, whereas Klintschar et al. [23] found a higher frequency of low-expressing alleles in male infants with SIDS. According to the pooled results of published research, the aim of this meta-analysis was to investigate the potential association between MAOA promoter VNTR polymorphism and the risk of SIDS, in an attempt to settle the debate and provide evidence for further research on the etiology of SIDS.

## Methods

### Search strategy

A systematic literature search was carried out in several accessible electronic databases, including PubMed, the Cochrane Library, Embase, MEDLINE, Chinese Biological and Medicine Disc, China Science and Technology Journal Database, China National Knowledge Infrastructure, Weipu Database, and Wanfang Database. Because of the language limitation, only literature in English and Chinese was selected. All research published before December 1, 2019, was collected by two independent reviewers (Q.Z. and D.G.) using key words including “sudden infant death syndrome,” “sudden infant death,” “unexpected infant death,” “MAOA,” “MAO-A,” “monoamine oxidase A,” “polymorphism,” and “susceptibility.” There was no restriction on the publication status.

### Inclusion and exclusion criteria

Original research regarding the potential association between genotype variants of MAOA gene promoter polymorphism and SIDS susceptibility was taken into consideration. All studies enrolled in the current meta-analysis were selected according to strict inclusion and exclusion criteria.

The inclusion criteria were as follows: (1) the studies were originally published as case–control studies; (2) the studies were on the correlation between MAOA gene promoter polymorphism and the risk of SIDS; (3) the infants enrolled in the studies were diagnosed with SIDS according to systematic autopsy and strict guidelines (e.g., the San Diego definition); and (4) the number of infants with specific gene variants was presented as a real number instead of a statistical analysis result.

The exclusion criteria were as follows: (1) reviews, case reports, and case series; (2) non-case–control studies or animal experiments; (3) studies on other diseases or sudden death in adults; (4) duplicate studies; (5) studies without original data or studies reporting the number of genotypes in mixed genders; and (6) studies with unexplained data (e.g., the number of X-chromosomes analyzed in a population of females was presented as an odd number).

### Data extraction and quality control

Data of the enrolled studies were collected by two independent reviewers (Q.Z. and D.G.) and verified by a third reviewer (Y.Z.) to reach a consensus. Basic information on each study was also collected, including the last name of the first author, year of publication, country and ethnicity of the subjects, methods of genotyping, and the age and number of infants. The quality of each study was assessed using the risk-of-bias tool (Cochrane USA, Washington, DC, USA). Potential bias was also estimated, including random sequence generation, allocation concealment, blinding of participants and personnel, blinding of outcome assessment, incomplete outcome data, selective reporting, and other bias. Bias was assessed and classified into three levels (low risk, high risk, and unclear risk of bias) and visualized in RevMan v.5.8 software (Cochrane Collaboration).

### Statistical analysis

Systematic meta-analysis was carried out using RevMan v.5.8 (Cochrane Collaboration) according to the data extracted from the included studies. To detect the existence of a single allele and the risk of SIDS, each VNTR variant was examined in each gender independently by comparing with the pooled results of other alleles. A comparison between the low-MAOA-expression allele (2R and 3R) and the high-MAOA-expression allele (3.5R and 4R) was also conducted to test the potential correlation between the risk of SIDS and the level of serotonin related to the VNTR variants. It should be noted that the 5R allele was not included in this comparison because of its rare existence in the whole populations and the related unclear serotonin level. In this meta-analysis, Higgins *I*^2^ was used to assess the heterogeneity between the studies. An *I*^2^ value less than 50% (or *P* > 0.05) suggests significant heterogeneity, suggesting the use of a fixed-effective model to summarize the data. Conversely, an *I*^2^ value greater than 50% (or *P* < 0.05) indicates unacceptable heterogeneity between the studies, highly suggesting the use of a random-effects model. The odds ratio (OR) of the pooled data was calculated using the 95% confidence intervals (CIs), and a *Z*-test was carried out to determine the significance of the results. A *P* value of < 0.05 was considered to represent statistical significance.

This systematic review and meta-analysis were conducted following the guidelines of preferred reporting items for systematic reviews and meta-analyses (PRISMA). No specific ethical approval from an institutional review board was required for this study as it used data from published literature. This meta-analysis has been prospectively registered on PROSPERO with the identification number of CRD42020169712 (https://www.crd.york.ac.uk/prospero/display_record.php?ID=CRD42020169712).

## Results

### Literature search

**Fig. 1:**
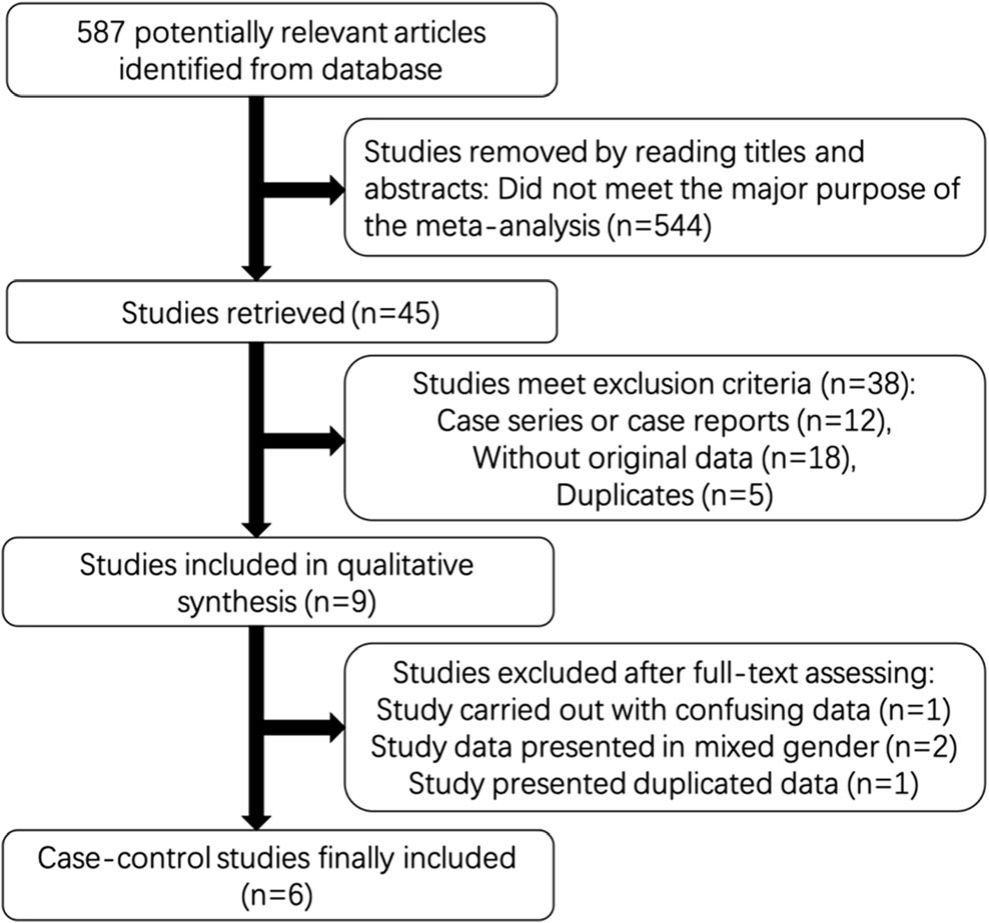
Flowchart of the literature search process

In total, 587 studies were pooled from the literature using a rough search of the databases mentioned above. After title screening, 141 studies remained on the potential association between MAOA promoter VNTR polymorphism and the risk of SIDS. Then, 45 articles were selected according to contents of the abstracts. After reading the full texts, 38 studies meeting the exclusion criteria were excluded, including 18 studies without original data, 5 duplicates, and 12 case series or case reports. In addition, a study by Filonzi et al. [17], published in 2009, was excluded because it presented an inappropriate elaboration of allelic frequencies. Although this error has been pointed out by Klintschar et al. [24] and clarified by Filonzi et al. in 2011 [17] in their following publication, the number of infants with different genotypes was presented without gender distinction, which introduced potential bias to the results because MAOA is a gene located on the X-chromosome. Additionally, another study by Marzano et al. [25] was excluded for the same reason. Besides, the data from Klintschar et al. [24] in 2010 is included in their later study [23], whereas their third paper [26] reported new data. Thus, the study by Klintschar et al. [24] in 2010 was excluded and the other two studies were retained. Finally, a total of six independent case–control studies were selected in this meta-analysis. A flow chart depicting the process of inclusion in this study is shown in Fig. 1.

### Characteristics of the studies

A total of six independent case–control studies [21–23, 26–28], including 1022 SIDS victims and 1150 controls, were selected in this meta-analysis. Among the included subjects, 401 females were classified into the case group and 416 females were classified into the control group. Thus, the total number of X-chromosomes analyzed in all the included studies was 1423 and 1566 in the case group and the control group, respectively. To clarify, Gross et al. [28] presented a pooled control group including the uSID and healthy individuals examined in their study, as well as the data from the study of Courts et al. [22] and Klintschar et al. [23]. To avoid potential bias, the number of control group examined in the study of Gross et al. was calculated by excluding those in the other two studies (Courts et al.: 280 controls, including 109 females and 171 males; Klintschar et al.: 260 controls, including 99 females and 151 males) from the pooled controls (585 controls, including 227 females and 358 males). Accordingly, there were 45 controls examined in the study of Gross et al., including 26 males and 19 females, which matches the framework of their enrolled controls (including 51 uSID cases and 42 healthy individuals) considering the possibility of PCR failures.

All studies were published between 2010 and 2014, and all of them were performed on Caucasian populations except for one on a mixed population of Caucasian and Caucasian Coriell subjects [27]. In exception of one study conducted using existing datasets [27], polymerase chain reaction was used as the genotyping method in all the other studies. All subjects included in the studies were 0–1 years of age (Table 1).

**Table 1:** Characteristics of all studies included in the meta-analysis.

Author [Ref]	Year	Country	Ethnicity	Study design	Genotyping method	*Characteristics of SIDS subjects	Characteristics of control subjects	No. (Case/Control)		
								No. of total individuals	No. of female individuals	No. of X chromosomes analyzed
Courts et al. [29]	2013	Germany	Caucasian	^†^RCC	^‡^PCR	Formalin fixed paraffin embedded samples, diagnosed according to “San Diego” definition, (age: 46–154 postnatal days)	Sex matched controls, biopsy material taken from the psoas major muscle* (153 males, 100 females), voluntarily donated saliva and blood samples (21 males, 9 females)	142/280	52/109	194/389
Gross et al. [24]	2014	Germany	Caucasian	^§^MCC	PCR	Diagnosed according to the San Diego definition (age: 2–51 weeks, mean: 20 week)	uSIDS and healthy individuals aged between 20–50 years old	261/45	107/19	368/64
Klintschar et al. [19]	2012	Germany	Caucasian	RCC	PCR	Aliquots of blood or tissue samples, 2–51 weeks, 19 weeks	Healthy young white subjects who had survived the critical time span of the first year without SIDS (age: 18–30 years; mean: 25)	156/260	57/99	213/359
Klintschar et al. [22]	2013	Germany	Caucasian	RCC	PCR	Aliquots of blood or tissue samples, 2–51 weeks, 19 weeks	Healthy young white subjects who had survived the critical time span of the first year without SIDS (age: 18–30 years; mean: 25)	128/138	50/58	178/196
Opdal et al. [30]	2013	Norway	Caucasian	RCC	PCR	Diagnosed according to the Nordic SIDS study criteria and the San Diego definition (age: 0.3–167 week, mean: 16 weeks, including 6 infants <2 weeks and 13 infants >1 year), long QT syndrome were excluded	Adult deceased controls (age: 11–91 years, median: 50 years, including 4 controls <18 years), randomly selected from autopsy, died from violent death, intoxication or disease (mainly ischaemic heart disease)	171/335	68/92	239/427
Paterson et al. [23]	2013	the USA	Caucasians and Caucasian Coriell	RCC	the San Diego SIDS Dataset	^∫^NA	NA	139/92	55/39	194/131

^†^RCC, retrospective case-control study

^§^MCC, multi-center case-control study

^‡^PCR, polymerase chain reaction

^∫^NA, not available

*Cases with potentially confounding alterations of autonomic and central nervous system were excluded

### Quality of the included studies and publication bias

**Fig. 2:**
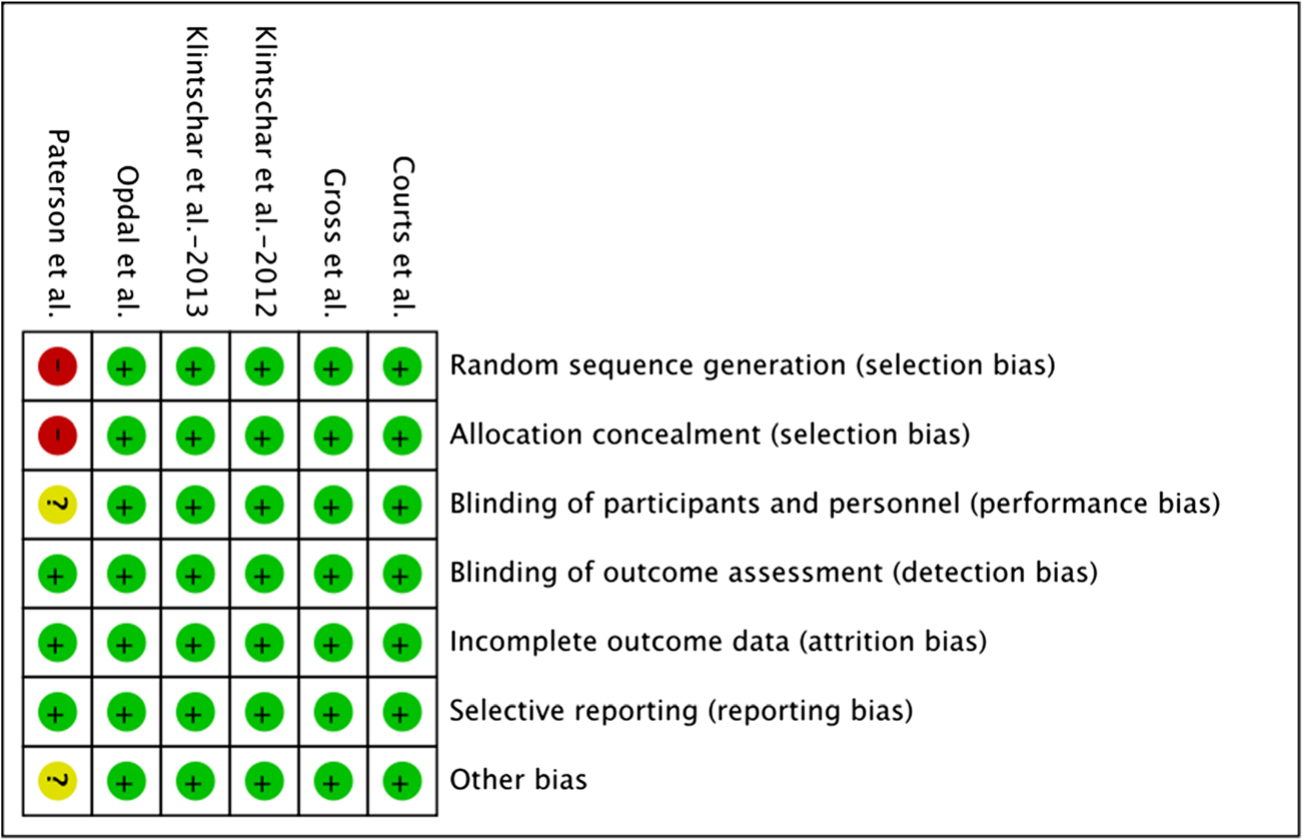
Risk of bias graph. A: Review authors’ judgments about each risk of bias item for each included study; B: review authors’ judgments about each risk of bias item presented as percentages across all included studies

The potential bias of each study was systematically assessed according to Cochrane’s risk-of-bias tool (Fig. 2). As detailed inclusion criteria of the subjects were not clearly presented in one study, potential selection bias tended to exist in this study. In general, the quality of the included studies was good since other studies did not exhibit potential risk of bias.

**Fig. 3:**
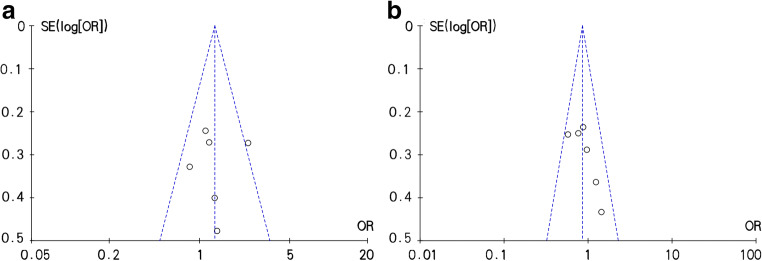
Publication bias analysis by funnel plots. A: male populations; B: female populations. SE, standard error; OR, odds ratio

According to the Egger test and funnel plot results, no significant publication bias was observed in comparison between low-MAOA-expression alleles (2R+3R) and high-MAOA expression alleles (3.5R+4R) (Fig. 3). Other comparisons did not exhibit significant publication bias as well.

### Quantitative data synthesis

In total, five variants of MAOA gene promoter polymorphism were estimated in this meta-analysis: 2R, 3R, 3.5R, 4R, and 5R. The number of subjects with different alleles and genotypes is shown in Table 2. It is worth mentioning that in the study of Gross et al. [28], the number of enrolled infants did not match the data presented in their table “Allele distribution of MAOA promoter polymorphism in this study’s SIDS cases and controls” (Number of X chromosomes analyzed: all SIDS, SIDS *n* = 368, control *n* = 812; male, SIDS *n* = 154, control *n* = 358; female, SIDS *n* = 214, control *n* = 454). Accordingly, they have successfully examined a total of 261 SIDS cases including 154 male cases and 107 female cases. Considering the possibility of failed PCR examinations, taking the number of all the infants enrolled in their study will obviously lead to some bias. Thus, this inconsistency was considered acceptable and only the number of examined cases and controls were extracted instead of all the enrolled infants. Besides, the numbers of subjects with different alleles and genotypes in control group were calculated by excluding those in the studies of Courts et al. [22] and Klintschar et al. [23] accordingly as described before.

**Table 2:** Original data of genotypes distribution extracted from the studies

**Author**	**No. of X chromosomes analyzed**		**Genotypes distribution of cases/controls**											
			**2R**		**3R**		**3.5R**		**4R**		**5R**		**Low expression (2R+3R)**	
	**Case**	**Control**	**Case**	**Control**	**Case**	**Control**	**Case**	**Control**	**Case**	**Control**	**Case**	**Control**	**Case**	**Control**
Alleles variant in male population														
Courts et al.	90	171	3	0	31	58	1	1	55	112	0	0	34	58
Gross et al.	154	26	0	0	52	7	3	0	95	18	4	1	52	7
Klintschar et al. (2012)	99	161	1	0	43	41	0	2	53	115	2	3	44	41
Klintschar et al. (2013)	78	80	0	0	29	33	2	1	46	45	1	1	29	33
Opdal et al.	103	243	0	0	39	85	0	7	64	149	0	2	39	85
Paterson et al.	84	53	2	1	23	12	-	-	57	40	2	0	25	13
Alleles variants in female population														
Courts et al.	104	218	4	0	28	94	1	4	71	119	0	1	32	94
Gross et al.	214	38	0	0	90	14	3	0	121	23	0	1	90	14
Klintschar et al. (2012)	114	198	0	1	36	74	0	1	77	122	1	0	36	75
Klintschar et al. (2013)	100	116	0	0	34	40	1	3	64	72	0	1	34	40
Opdal et al.	136	184	0	0	47	69	2	0	87	115	0	0	47	69
Paterson et al.	110	78	0	0	30	18	-	-	77	58	3	2	30	18
	**No. of female patients**		**2R/2R**		**3R/3R**		**3R/4R**		**3.5R/4R**		**4R/4R**		**4R/5R**	
	**Case**	**Control**	**Case**	**Control**	**Case**	**Control**	**Case**	**Control**	**Case**	**Control**	**Case**	**Control**	**Case**	**Control**
Genotype polymorphism in female population														
Courts et al.	52	109	2	0	9	26	10	41	1	4	30	37	0	1
Gross et al.	107	19	0	0	27	3	36	8	3	0	41	7	0	1
Klintschar et al. (2012)	57	99	0	1	7	16	22	41	0	1	27	40	1	0
Opdal et al.	68	92	0	0	7	17	33	35	2	0	26	40	0	0

**Fig. 4:**
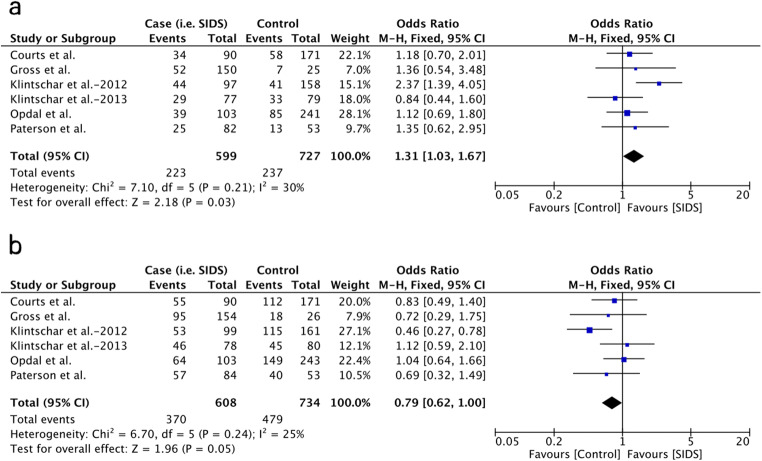
Forest plots of comparisons regarding allele variants in male infants. A: low-MAOA-expression alleles vs. high-MAOA-expression alleles in male infants; B: 4R allele vs. other alleles in male infants

Male infants with a 4R allele exhibited a tendency of reduced SIDS risk (OR = 0.79, 95% CI = 0.62–1.00, *P* = .05; Fig. 4), whereas those with other single alleles did not show significant association with SIDS risk. Regarding the possible correlation between the expression levels of MAOA and susceptibility to SIDS, the pooled results of the low-MAOA-expression allele (2R+3R) were also examined in comparison to the high-expression allele (3.5R+4R). The 5R allele was not included in the comparison as the function of 5R in the serum level of MAOA and 5-HT has not been confirmed yet. The results showed that the low-MAOA-expression alleles (2R+3R) are associated with the occurrence of SIDS (OR = 1.31, 95% CI = 1.03–1.67, *P* = 0.03; Fig. 4). No correlation was observed between SIDS and the 2R, 3R, 3.5R, and 5R alleles in male infants, and none of the allele or genotype variants was associated with SIDS in female patients. The detailed results of each comparison are shown in Table 3.

**Table 3:** Meta-analysis of the association between MAOA promoter polymorphism and sudden infant death syndrome risk

Comparing model	Overall analysis						Subgroup analysis, Caucasian population					
	No. of studies	Association results		Heterogeneity			No. of studies	Association results		Heterogeneity		
		^†^OR (95% ^§^CI)	*P* (Z-t)	*P* (Q-t)	I^2^ (%)	Model		^†^OR (95% ^§^CI)	*P* (Z-t)	*P* (Q-t)	*I*^2^ (%)	Model
Alleles variant in male population												
2R vs. others	6	4.16 (0.89, 19.36)	0.07	0.46	0	^‡^F	6	*7.10 (1.22, 41.22)*	*0.03*	0.85	0	F
3R vs. others	6	1.26 (0.99, 1.61)	0.06	0.23	27	F	6	*1.30 (1.01, 1.67)*	*0.04*	0.16	37	F
3.5R vs. others	5	0.63 (0.22, 1.85)	0.41	0.58	0	F	5	0.63 (0.22, 1.85)	0.41	0.58	0	F
4R vs. others	6	0.79 (0.62, 1.00)	0.05	0.24	25	F	6	*0.76 (0.59, 0.97)*	*0.03*	0.1	46	F
5R vs. others	6	1.03 (0.36, 2.92)	0.96	0.92	0	F	6	0.96 (0.33, 2.78)	0.93	0.95	0	F
Low expression vs. high expression*	6	*1.31 (1.03, 1.67)*	*0.03*	0.21	30	F	6	*1.37 (1.07, 1.76)*	*0.01*	0.1	46	F
Alleles variants in female population												
2R vs. others	6	4.77 (0.95, 23.95)	0.06	0.11	61	F	6	4.77 (0.95, 23.95)	0.06	0.11	61	F
3R vs. others	6	0.83 (0.67, 1.04)	0.1	0.19	32	F	6	0.83 (0.66, 1.04)	0.1	0.16	37	F
3.5R vs. others	5	0.91 (0.32, 2.54)	0.85	0.62	0	F	5	0.91 (0.32, 2.54)	0.85	0.62	0	F
4R vs. others	6	1.17 (0.94, 1.46)	0.15	0.38	6	F	6	1.18 (0.95, 1.48)	0.14	0.36	8	F
5R vs. others	6	0.75 (0.26, 2.23)	0.61	0.39	4	F	6	0.62 (0.19, 2.08)	0.44	0.42	0	F
Low expression vs. high expression*	6	0.86 (0.69, 1.07)	0.17	0.41	0	F	6	0.85 (0.68, 1.07)	0.16	0.36	8	F
Genotype polymorphism in female population												
2R/2R vs. others	4	2.84 (0.48, 16.72)	0.25	0.19	41	F						
3R/3R vs. others	4	0.74 (0.46, 1.18)	0.2	0.48	0	F						
3R/4R vs. others	4	0.85 (0.59, 1.22)	0.38	0.07	58	^∫^R						
3.5R/4R vs. others	4	1.21 (0.36, 3.99)	0.76	0.56	0	F						
4R/4R vs. others	4	1.34 (0.94, 1.90)	0.11	0.09	54	R						
4R/5R vs. others	4	0.70 (0.15, 3.31)	0.65	0.15	48	F						

^†^*OR*, odds ratio

^§^*CI*, confidence intervals

^‡^*F*, fixed-effective model

^∫^*R*, random-effective model

*The pooled alleles of 2R+3R (low-expression) vs. 3.5R+4R (high-expression), the rare 5R allele was excluded; results of statistic significant association are presented in italic

**Fig. 5:**
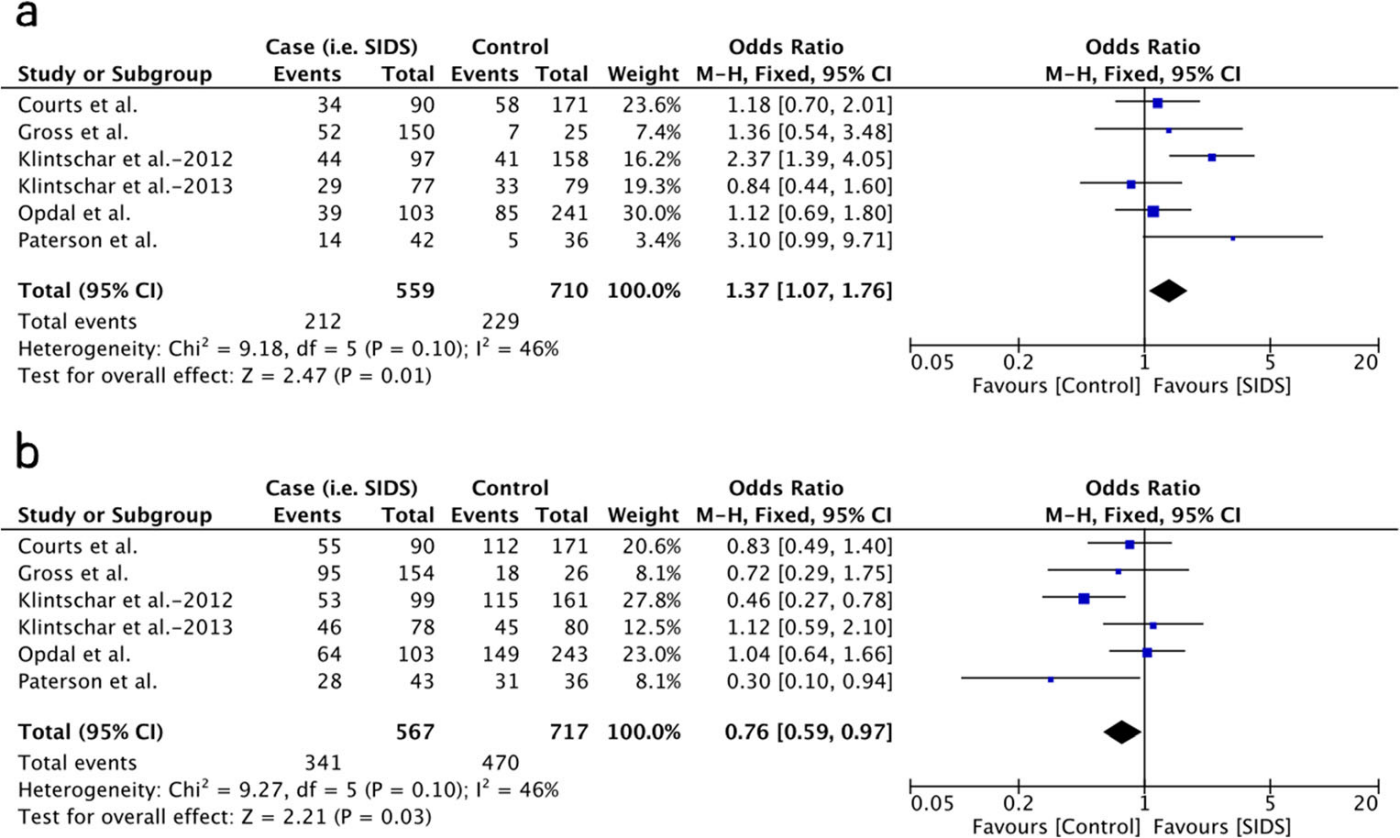
Forest plots of comparisons regarding allele variants in male Cacasian infants. A: low-MAOA-expression alleles vs. high-MAOA-expression alleles in male Caucasian populations; B: 4R allele vs. other alleles in male Caucasian populations

Considering the differences of genotype frequency among different populations, further comparisons were conducted excluding the Caucasian Coriell populations presented in the study of Paterson et al. [27] as most of the studies were performed on Caucasian infants. As shown in Table 3, a significantly increased risk of SIDS was observed in male Caucasian infants with the 2R allele (OR = 7.10, 95% CI = 1.22–41.22, *P* = 0.03) and 3R allele (OR = 1.30, 95% CI = 1.01–1.67, *P* = 0.04). A significant association was also observed in the low-MAOA-expression group, in which the results of 2R and 3R are pooled together (OR = 1.37, 95% CI = 1.07–1.76, *P* = 0.01; Fig. 5). The 4R allele, which is presented at a high frequency and exhibited a tendency with reduced SIDS risk in the whole male populations, was proved to be a protective factor against SIDS with statistic significance in male Caucasian infants (OR = 0.76, 95% CI = 0.61–0.94, *P* = 0.01; Fig. 5). Regarding the other two rare alleles, both 3.5R (OR = 0.63, 95% CI = 0.22–1.85, *P* = 0.41) and 5R (OR = 0.96, 95% CI = 0.33–2.78, *P* = 0.93) did not show a significant correlation to SIDS susceptibility in male Caucasian infants. None of the allele or genotype variants was associated with SIDS in female Caucasian patients.

### Sensitivity analysis

The effect of each individual study on the total results was estimated by removing the results of the studies one by one to observe the change that occurs in the overall results. After excluding the study of Gross et al. [28], the study with the largest population among the included studies, a statistically significant protective influence of 3R/3R polymorphism emerged among females (OR = 0.61, 95% CI = 0.37–0.98, *P* = 0.04). The stability of other comparisons conducted on male infants was not affected by removing any of the studies.

## Discussion

It has been reported in previous research so-called “triple-risk-hypothesis” that the development of SIDS is affected by the interaction between environmental challenges and the diverse genetic properties of the individuals as well as the developmental factors [3]. There were abundant combined interactions between the factors, which may result in the critical impairment of autonomous regulation in individuals, leading to SIDS [26, 27]. Thus, it is worth detecting a genetic alteration that could biologically affect the bearers in SIDS cases, which is also of great significance to proceed with the development of research on the etiology of SIDS.

To the authors’ knowledge, this meta-analysis is the first meta-analysis aiming to assess the potential correlation between MAOA promoter VNTR polymorphism and the risk of SIDS, and it is also the first meta-analysis discussing the association between gene polymorphisms and susceptibility to SIDS. By pooling the results of published studies with different sample sizes and applying subgroup analysis, reliable evidence was provided for the results. It was observed that the 2R and 3R alleles increased the risk of SIDS in Caucasian male infants, and that the presence of 4R decreases the risk of SIDS in male infants, whether Caucasian or mixed populations, which can be regarded as a protective factor. It was also confirmed that there is a correlation between the low-MAOA-expression allelic variants (2R and 3R alleles) and the development of SIDS in male infants, indicating potential correlations between low serotonin levels and the development of SIDS, which also coincided with previously published studies. However, it is worth mentioning that the study of Gross et al. [28], which was also included in our meta-analysis, presented their results pooled with two previously published studies and came to a conclusion denying the significant dependencies between SIDS and MAOA promoter polymorphisms, which was in stark contrast to our findings. Considering the relatively small number of controls enrolled in their study and the fact that uSID cases constitute half of the control group, which could not exclude the possibility of autonomic nervous system disturbances and correlated MAOA polymorphisms, heterogeneity was possible to exist between their study and the pooled studies. The current meta-analysis expanded the number of enrolled infants by including studies with good quality, and carried out heterogeneity examinations and sensitivity analysis test, which provided higher reliability to the results. Despite the difference of the results, there was a protective influence of 3R/3R polymorphism showed up in female populations during the sensitivity analysis which also could not be confirmed yet. Thus, further studies with larger populations are highly recommended in the future.

Previously published studies have proposed a subset of SIDS with a high serum level of serotonin, which may be related to 5-HT network abnormalities, suggesting that an increased serum serotonin level is a potential biomarker for the diagnosis of SIDS in forensic science [31]. A mechanism for 5-HT-related SIDS has also been hypothesized, indicating that the high-MAOA-expressing genotype of the promoter is related to enhanced vesicular serotonin catabolism, following increased serotonin reuptake driven by the dopamine transporter [17]. Metabolic and environmental factors also contribute to the lack of production of tryptophan, leading to a reduction in interneuronal serotonin release and consequently resulting in a higher serotonin level [17, 32]. Given that MAOA plays an important role in the inactivation process of serotonin and noradrenalin in the human brainstem, the findings of the higher frequency in male infants with SIDS carrying low-expression alleles of the MAOA promoter are, thus, supported by theoretical evidence, with the correlation between 5-HT level and SIDS being of great potential for further investigation.

Besides, male infants were reported to exhibit a 30–50% higher risk of SIDS comparing to female infants [33, 34], with the hypothesis that functional genes located on the X-chromosome are involved [35]. As a X-chromosome located gene reported to be associated with the risk of SIDS, the polymorphism of the MAOA promoter may be partially involved in the different percentages of occurrence of SIDS between genders. Although female homozygotes should also exhibit the same trend of susceptibility to SIDS, this study showed there is no association between MAOA promoter polymorphism and the risk of SIDS in female infants. Possible explanations, however, include the limited number and relatively small sample sizes of the included studies and the fact that external factors may affect the risk of disease speculated by genotype. Thus, according to the limited information provided by the current meta-analysis, it is impossible to investigate the dominant or recessive character of MAOA alleles in the development of SIDS.

Given the triple-risk-hypothesis, SIDS risk has been proved to be correlated with quite different genetic predispositions because different evidence could be integrated into various hypotheses for explanation of the SIDS etiology. Thus, gene polymorphisms like MAOA locus, which may not constitute a lethal factor, are better to be regarded as a role disposes to SIDS and added to the genetic background influence in a triple-risk model. Limited by the available information in the enrolled studies, heterogeneity raised by environmental and developmental factors such as age, temperature, sleeping posture, and feeding patterns could not be excluded, although it did not show up in this current meta-analysis. To get a better grasp of the possible etiology, studies with more detailed information will be encouraged in the future.

This meta-analysis included some limitations that should be addressed here. First of all, given that only individuals of the same race were included in the studies, associations could be examined only in Caucasian infants, and thus studies on populations with other ethnicities are highly recommended in the future. However, this limitation might have been useful for the current study because it helped exclude the influence of hybrid genes introduced by mixed populations, which also exhibited low heterogeneity between the studies in the meta-analysis. Second, publication bias tends to exist in research, although none was observed in this study. Except for unpublished studies with negative results, it is possible that more studies published in other languages exist. Third, the number of studies included in the current meta-analysis is relatively small compared with other studies, and there are more published studies investigating the correlation between the polymorphism of MAOA promoter and sudden infant death risk. However, this study is designed with strict inclusion and exclusion criteria in the process of study selection. Firstly, considering that the MAOA gene is an X-chromosome located gene, the studies presented in mixed-gender would obviously introduce some potential bias as the frequency of the female subjects could be counted twice. Secondly, there were odd numbers presented in some studies regarding the total alleles of the female populations, which we regarded them as “unexplained” and were further excluded due to unreliable data. Thirdly, some studies reported the updated data based on their previously published work, which included repeated populations. Thus, only the latest study or the study with the largest populations was included in this current work. In order to ensure the accuracy and scientificity of the meta-analysis, studies with the problems mentioned above have to be excluded. To our knowledge, there were no more published studies could be included. We believe it is necessary to complete the study with strict inclusion criteria, and we hope this meta-analysis could provide a reference for further researches to avoid the problems mentioned above. Besides, as gene polymorphism may combine with various factors and contribute to the development of SIDS, it is very important to perform further subgroup analyses. However, this was not possible here because of the restrictions due to the limited information of the subjects in the included studies. Thus, it is highly recommended to perform more systematically designed studies with detailed and scientifically presented data in the future.

## Conclusion

According to this meta-analysis, low-expression MAOA promoter VNTR alleles (2R and 3R) are associated with an increased risk of SIDS compared to high-expression alleles (3.5R and 4R) in male Caucasian infants, and the existence of the 4R allele could be regarded as a protective factor against the development of SIDS in male Caucasian infants. This study also suggested that abnormalities in the serotonergic system and nervous system disturbances may contribute to the development of SIDS. In conclusion, it is recommended to perform more systematically designed studies with detailed data in the future.

## Abbreviations

CIs: confidence intervals
MAOA: monoamine oxidase A
OR: odds ratio
PRISMA: Preferred Reporting Items for Systematic Reviews and Meta-Analyses
SIDS: sudden infant death syndrome
VNTR: variable number tandem repeat
5-HT: 5-hydroxytryptamine
